# Deletion of *steD* gene and *yejAE* operons in LPS-deficient *Salmonella* elicits strong t cell immunity and complete protection against homologous serotypes

**DOI:** 10.3389/fmicb.2026.1827967

**Published:** 2026-06-17

**Authors:** Guodong Zhou, Zhihui Zhang, Qingyu Wang, Zhili Luo, Shengliang Cao, Yubao Li

**Affiliations:** 1 College of Agriculture and Biology, Liaocheng University, Liaocheng, Shandong, China; 2 Shandong Key Laboratory of Applied Technology for Protein and Peptide Drugs, Liaocheng University, Liaocheng, Shandong, China; 3 College of Pharmaceutical Sciences and Food Engineering, Liaocheng University, Liaocheng, Shandong, China

**Keywords:** immune evasion, lipopolysaccharides (LPS), *Salmonella* Typhimurium, SteD, T cell immunity, *yejAE*

## Abstract

Non-typhoidal *Salmonella* (NTS) infections are a major cause of foodborne illness worldwide, and rising antimicrobial resistance underscores the need for effective vaccines. Live attenuated *Salmonella* strains have potential, but developing an effective vaccine remains a challenge. In this study, we built on the previously constructed attenuated *Salmonella* Typhimurium strain RAST002, which carries deletions of *manA* and *steD*, by introducing a *yejAE* operon deletion to generate RAST003, aiming to further enhance immune responses. Oral immunization with this single strain induced robust systemic IgG and mucosal sIgA responses, significantly increased CD4^+^ and CD8^+^ T cell frequencies, and markedly elevated IFN-γ production, indicating a dominant Th1-type cellular immune response, alongside increased IL-2 and TNF-α levels supporting overall T cell activation. Immunized mice were fully protected against homologous *S*. Typhimurium challenge and exhibited significantly improved survival following heterologous *S*. Enteritidis exposure. These results demonstrate that combining LPS deficiency with targeted deletions of immune evasion genes generates a single, highly immunogenic *Salmonella* strain capable of inducing broad, protective immunity, providing a promising strategy for NTS vaccine development.

## 1 Introduction

*Salmonella* Typhimurium (*S*. Typhimurium) is recognized globally as a major non-typhoidal *Salmonella* (NTS) serovar responsible for foodborne illnesses (Ferrari et al., 2019; Bearson, 2022; Chousalkar and Willson, 2022). NTS infections typically manifest as self-limiting gastroenteritis, characterized by symptoms such as diarrhea, abdominal pain, and nausea (Fierer, 2022). However, in individuals with weakened immune systems, *Salmonella* infection can lead to more severe invasive conditions, such as septicemia and endocarditis (Fierer, 2022). While *Salmonella* species are found in various environmental sources, animals, particularly livestock, are the primary reservoirs. Human infections primarily occur through the consumption of contaminated food products, especially poultry, eggs, and meat, which are contaminated through the feces of infected animals (Ferrari et al., 2019; Bearson, 2022; Chousalkar and Willson, 2022). *S.* Typhimurium, frequently isolated from a variety of livestock species such as pigs, cattle, and poultry, remains a key player in human infections (Ferrari et al., 2019; Chen et al., 2025). Given the widespread presence of *S*. Typhimurium in food animal production, numerous countries have implemented control measures, including vaccination programs, to mitigate the risk of transmission to humans.

Vaccination of livestock, particularly those with significant economic value, is regarded as one of the safest and most efficient measures to prevent *Salmonella* transmission to humans. Live attenuated vaccines are often preferred because they stimulate both cellular and humoral immune responses, offering durable immunity (Tennant and Levine, 2015). Numerous live attenuated vaccines are currently available for use in pigs and poultry worldwide. In Australia, for example, a live attenuated *S*. Typhimurium vaccine has been developed by disrupting the *aroA* gene via the insertion of the Tn10 transposon (Alderton et al., 1991). Moreover, IDT Biologika has licensed two auxotrophic *Salmonella* strains, Salmovac440 for chickens and Salmoporc for pigs, both of which require histidine and adenine supplementation (Schmidt et al., 2021; Springer et al., 2021). Recent whole-genome sequencing (WGS) studies have revealed that Salmovac440 is attenuated by six single nucleotide polymorphisms (SNPs), which significantly reduce the strain’s virulence (Tang et al., 2019). However, the mutations caused by these SNPs are prone to reversion, which could potentially restore the strain’s original virulence. This highlights the need for the identification of novel gene deletions as potential candidates for generating more stable live attenuated vaccines, which would mitigate the risk of reversion and enhance the overall safety and efficacy of *Salmonella* vaccines.

The deletion of virulence factors has become a common approach in the development of live attenuated vaccines, as it helps reduce bacterial pathogenicity while maintaining immunogenicity (Yin et al., 2015; Lin et al., 2017; Zhen et al., 2026; Zhong et al., 2025). Among these virulence factors, lipopolysaccharide (LPS) plays a critical role in *Salmonella* virulence, enabling successful infection and persistence within the host (Crhanova et al., 2011). Disrupting LPS biosynthesis leads to several impairments in *Salmonella* physiology, including reduced growth, motility, intestinal colonization, and resistance to immune defenses such as serum bactericidal activity and macrophage-mediated killing (Shaio and Rowland, 1985; Nevola et al., 1987; Nagy et al., 2006; Crhanova et al., 2011; Kong et al., 2011; Klein and Raina, 2019). Furthermore, LPS modification enhances the surface exposure of conserved outer membrane proteins (OMPs), which improves antigen presentation and boosts the induction of protective immune responses (Domínguez-Medina et al., 2020). In addition to LPS, *Salmonella* employs immune evasion strategies to escape host immune surveillance. One such mechanism involves the SteD protein, encoded by the *Salmonella* pathogenicity island 2 (SPI-2), which suppresses T cell activation by downregulating the expression of MHC class II, CD86, and CD97-key molecules involved in antigen presentation and T cell activation (Bayer-Santos et al., 2016; Cerny et al., 2021; Godlee et al., 2022). Deleting the *steD* gene may enhance T cell-mediated immunity, particularly CD4^+^ T cell responses, and improve protection against *Salmonella* (Allen et al., 2024). Similarly, the *yejAE* operon has been shown to influence the MHC class I antigen presentation pathway, and its deletion could enhance CD8^+^ T cell responses (Qimron et al., 2004; Hummel et al., 2005). Therefore, the combined deletion of *steD* and *yejAE* in an LPS-deficient *Salmonella* strain may have the potential to significantly improve both CD4^+^ and CD8^+^ T cell responses, offering a promising strategy to enhance vaccine efficacy and cross-serotype protection.

In this study, we aimed to enhance the immunogenicity and protective capacity of an LPS-deficient *Salmonella enterica* serovar Typhimurium strain by introducing targeted deletions of the *steD* and *yejAE* genes. The immunogenicity of the resulting mutant strain, designated RAST003, was evaluated in BALB/c mice. Following immunization, serum IgG and vaginal lavage IgA titers were measured, and functional antibody responses, along with *Salmonella*-specific T cell activation, were assessed. Furthermore, the protective efficacy of this vaccine candidate was examined against homologous *Salmonella* serovars post-immunization. Overall, this study aimed to develop a cost-effective, practical, and effective live attenuated vaccine capable of inducing strong immune responses and providing robust protection against homologous *Salmonella* infections.

## 2 Materials and methods

### 2.1 Ethics statement

All BALB/c mice used in this study were sourced from Jinan Pengyue Laboratory Animal Breeding Co., Ltd. The animal procedures were carried out in full compliance with the institutional guidelines of Liaocheng University, under the approved protocol number AP2025031708. The study was reviewed and approved by the Institutional Animal Care and Use Committee (IACUC) of Liaocheng University. For the animal experiments, all efforts were taken to minimize the suffering of the animals.

### 2.2 Bacterial strain, plasmids, media, and growth conditions

The plasmids and strains used in this study are listed in Table 1. *Salmonella* Typhimurium ATCC 14028 was grown at 37°C in Luria-Bertani (LB) broth. This strain served as the parental background for constructing the attenuated live vaccine candidates and was also used as the challenge strain in mouse infection experiments. The *S*. Enteritidis field isolates SE1, SE2, and SE3 were propagated under identical conditions. These isolates were subsequently employed in heterologous challenge studies to assess cross-serotype protection. Bacterial strains *E. coli* χ7213 and the suicide vector plasmid pRE112 were obtained from Baosai Biotechnology (China). The pRE112-derived suicide plasmid carrying the *yejAE* operon flanking regions was constructed based on pRE112 and used to introduce a *yejAE* deletion into RAST002. When required, the medium was supplemented with 50 μg/mL Ampicillin (Amp), 50 μg/mL Chloramphenicol (Cm), 50 μg/mL 2,6-diaminopimelic acid (DAP). In addition, D-mannose (0.2% wt/vol) and sucrose (5% wt/vol) were added at the indicated concentrations.

**TABLE 1:** Bacterial strains and plasmids used in this study.

Strains and plasmid	Characteristics	Sources, references, or function
E. coli strains		
DH5α	For recombinant plasmid construction	Lab stock
χ7213	*thi-1 thr-1 leuB6 fhuA21 lacY1 glnV44 asdA4 recA1 RP4 2-Tc:Mu pir*	Lab stock
S. Typhimurium		
ATCC14028 RAST001 RAST002 RAST003	Wild type; virulent A derivative strain of ATCC14028 with a deletion of the *manA* gene. A derivative strain of ATCC14028 in which both the *manA* and *steD* genes have been deleted A derivative strain of ATCC14028 in which *manA*, *steD*, and *yejAE* have been deleted	(Zhou et al., 2025b) This study
S. Enteritidis		
SE1 SE2 SE3	Wild type; highly virulent strain; Isolated from poultry in different regions of Shandong Province, China	(Zhou et al., 2025b)
Plasmids		
pRE112 pRE112-*yejAE*	Bacterial allelic exchange vector with SacB; Contains lambda pir-dependent R6K replication origin; requires lambda pir-containing bacteria strain; Cm^r^ A pRE112-derived suicide plasmid containing *yejAE* flanking regions for gene deletion	Lab stock This study
pMD19-T	Cloning vector; Amp^r^	TaKaRa

Amp^r^, Ampicillin resistance; Cm^r^, Chloramphenicol resistance.

### 2.3 Molecular and genetic procedures

Suicide vectors and primers used in this study are listed in Tables 1, 2, respectively. DNA fragment assembly was performed using the Seamless Cloning Kit (C116, Vazyme), according to the manufacturer’s instructions. To generate unmarked gene deletions in *S*. Typhimurium (ATCC14028), SacB-based sucrose counter-selectable suicide vectors were utilized (Zhou et al., 2025b). Deletion constructs were created by amplifying and purifying two homologous regions flanking the target gene, which were then fused via overlap extension PCR and ligated into the pRE112 plasmid. Conjugative transfer of recombinant plasmids into *S*. Typhimurium was achieved using the donor strain χ7213. Transconjugants were selected on LB agar containing chloramphenicol and lacking DAP. The second homologous recombination event, which led to the excision of the integrated suicide vector, was selected by plating colonies on LB agar supplemented with 5% sucrose and without sodium chloride, followed by incubation at 25°C. Successful deletion mutants were identified by colony PCR and verified through Sanger sequencing.

**TABLE 2:** The primers information.

Primer name	Sequences (5′–3′)	References
*yejAE*-1	aagcttcttctagaggtaccTACTGGTCACTATGGATTTTCCT	This study
*yejAE*-2	agcgattgaccgtcaaacacGGAGCAAATCGTTAACATCAG	
*yejAE*-3	GTGTTTGACGGTCAATCG	
*yejAE*-4	atgaattcccgggagagctcGCTGGAGCGATTTCAGG	
*yejAE*-F	TACTGGTCACTATGGATTTTCCT	
*yejAE*-R	GCTGGAGCGATTTCAGG	
*manA*-F	ACTTAATTCGACACCGGC	(Zhou et al., 2025b)
*manA*-R	AACAAGTAGTACTCGGTATCGT	
*steD*-F	GGTCCATTATTCTCTGCAAAC	
*steD*-R	GGAAATCACAAGGGGTAGTC	
pRE112-F	GTACTGCGATGAGTGGCAGG	
pRE112-R	TCGTGTTGAGGCCAACGC	
M13-F	GATGTGCTGCAAGGCGATTA	
M13-R	TTATGCTTCCGGCTCGTATG	

Lowercase letters denote homologous sequences designed for overlap PCR and recombination with the plasmid backbone. Primer pairs yejAE-1/yejAE-2 were used to amplify the upstream homologous arm of the deleted *yejAE* operon, and primers yejAE-3/yejAE-4 were used to amplify the downstream homologous arm. The two homologous arms were subsequently joined by overlap PCR to generate the deletion construct.

### 2.4 Immunization of mice

Female BALB/c mice (6–8 weeks old) were obtained from Jinan Pengyue Laboratory Animal Breeding Co., Ltd. and randomly assigned into three experimental groups: Group 1 (PBS control, *n* = 33), Group 2 (RAST002, *n* = 33), and Group 3 (RAST003, *n* = 33). Mice in the PBS control group were orally administered 20 μL of PBS without bacteria, while mice in Groups 2 and 3 received 20 μL of PBS containing 1.0 × 10^9^ CFU of the corresponding strain. A homologous booster immunization with the same dose of the same strain was administered orally on day 21. Following immunization, animals were monitored daily for general health and clinical signs.

### 2.5 Sample collection for analysis of antibody responses

To evaluate systemic and mucosal antibody responses, blood and vaginal lavage samples were collected from immunized mice at 7 days after the primary immunization and 7 days after the booster immunization. For serum preparation, blood was collected from mice and allowed to clot at room temperature, followed by centrifugation at 4,000 × g for 10 min to obtain serum, which was stored at -80°C until analysis. For vaginal lavage collection, mice were gently restrained and the vaginal cavity was flushed with sterile PBS using a micropipette. The recovered lavage fluid was collected into sterile tubes and clarified by centrifugation at 4°C to remove debris. The resulting supernatants were harvested and stored at -80°C for subsequent measurement of antigen-specific IgA levels by ELISA.

Outer membrane protein (OMP) extraction was performed as previously described and used for antibody detection and cellular immune response assays (Kang et al., 2002). Briefly, periplasmic proteins were removed by lysozyme-osmotic shock, cells were disrupted, total envelopes were collected by ultracentrifugation, and the outer membrane fraction was obtained after Sarkosyl treatment of the envelopes. The resulting pellet was resuspended and used as the OMP preparation (ST-OMPs and SE-OMPs).

### 2.6 Determination of antibody responses by enzyme-linked immunosorbent assay (ELISA)

ELISAs were performed in triplicate to determine antigen-specific IgG responses in mouse sera and IgA responses in vaginal lavage samples. Briefly, serum samples and vaginal lavage fluids collected at 7 days after the primary immunization and 7 days after the booster immunization were used for antibody analysis. An initial dilution of 1:200 was prepared for sera and 1:10 for vaginal lavage samples. Subsequently, 2-fold serial dilutions were performed and added in triplicate to 96-well Nunc MaxiSorp plates pre-coated with the corresponding antigens. After incubation and washing, bound antibodies were detected using HRP-conjugated goat anti-mouse IgG or IgA secondary antibodies at appropriate dilutions. Color development was performed using TMB substrate, and the reaction was stopped with stop solution. Absorbance was measured at 450 nm using a microplate reader, and antibody titers were calculated based on the endpoint dilution.

### 2.7 Splenocyte proliferation and cytokine secretion assays

Splenocyte proliferation and cytokine response assays were performed as described previously with minor modifications. Briefly, mice were euthanized at 7 days after the booster immunization, and spleens were aseptically collected. Single-cell suspensions were prepared by gently pressing spleens through a 70 μm cell strainer, followed by red blood cell lysis using Red Blood Cell Lysis buffer. After washing, splenocytes were resuspended in RPMI-1640 complete medium and seeded into 96-well plates. Cells were stimulated with 10 μg/mL of ST-OMPs or SE-OMPs, while unstimulated cells served as negative controls. After incubation at 37°C with 5% CO_2_, splenocyte proliferation was assessed using a Cell Counting Kit-8 (CCK-8) assay according to the manufacturer’s instructions, and the stimulation index (SI) was calculated as the ratio of the mean absorbance of antigen-stimulated wells to that of unstimulated control wells.

In parallel, splenocytes were plated in duplicate at a density of 5 × 10^5^ cells per well in 96 well plates and stimulated with ST-OMPs or SE-OMPs. RPMI 1640 medium alone served as the negative control. Culture supernatants were collected at defined time points for cytokine analysis. Supernatants harvested at 24 h were used to determine IL-2 and TNF-α levels to evaluate early T cell activation, whereas supernatants collected at 72 h were used to measure IFN-γ, IL-4, and IL-17A to assess antigen specific Th1, Th2, and Th17 responses. Cytokine concentrations in the collected supernatants were quantified using commercial ELISA kits according to the manufacturers’ instructions. All assays were performed in triplicate.

### 2.8 Flow cytometric analysis of T cell subsets

T cell subsets were analyzed as previously described (Zhou et al., 2025b). Briefly, splenocytes were harvested one week after the final immunization and resuspended in PBS after red blood cell lysis. Fc receptors were blocked with anti-mouse CD16/32 antibody to prevent non-specific binding. Cells were subsequently incubated with a viability dye to exclude dead cells, followed by staining with fluorochrome-conjugated antibodies, including APC-anti-CD4 for CD4^+^ T cells and PE-anti-CD8 for CD8^+^ T cells. After washing to remove unbound antibodies, cells were resuspended in PBS and analyzed by flow cytometry. Lymphocytes were first gated based on forward and side scatter properties, and CD4^+^ and CD8^+^ T-cell populations were subsequently identified for analysis (Teran et al., 2011).

### 2.9 Challenge experiments

Mice were challenged orally with virulent *Salmonella* strains as appropriate for each experimental group. Briefly, animals were administered approximately 100 × LD_50_ of either the wild-type *S*. Typhimurium strain ATCC14028 or one of the field *S*. Enteritidis isolates (SE1, SE2, or SE3) by oral gavage. After challenge, mice were monitored daily for 21 consecutive days, and survival was recorded throughout the observation period for subsequent analysis.

### 2.10 Statistical analysis

All data are presented as mean ± standard deviation (SD). Statistical analyses were conducted using GraphPad Prism software (version 9.5; GraphPad Software Inc., San Diego, CA). Differences among groups were evaluated using one-way analysis of variance (ANOVA) followed by Tukey’s multiple comparison test. The survival after the challenge was compared using the log-rank (Mantel-Cox) test. A *p* ≤ 0.05 was considered statistically significant. The level of significance is indicated as follows: *p* < 0.05 (*), *p* < 0.01 (**), *p* < 0.001 (***), and *p* < 0.0001 (****). *P* > 0.05 were considered not significant (n.s).

## 3 Results

### 3.1 Construction of the live attenuated *S*. Typhimurium vaccine candidates

**FIGURE 1:**
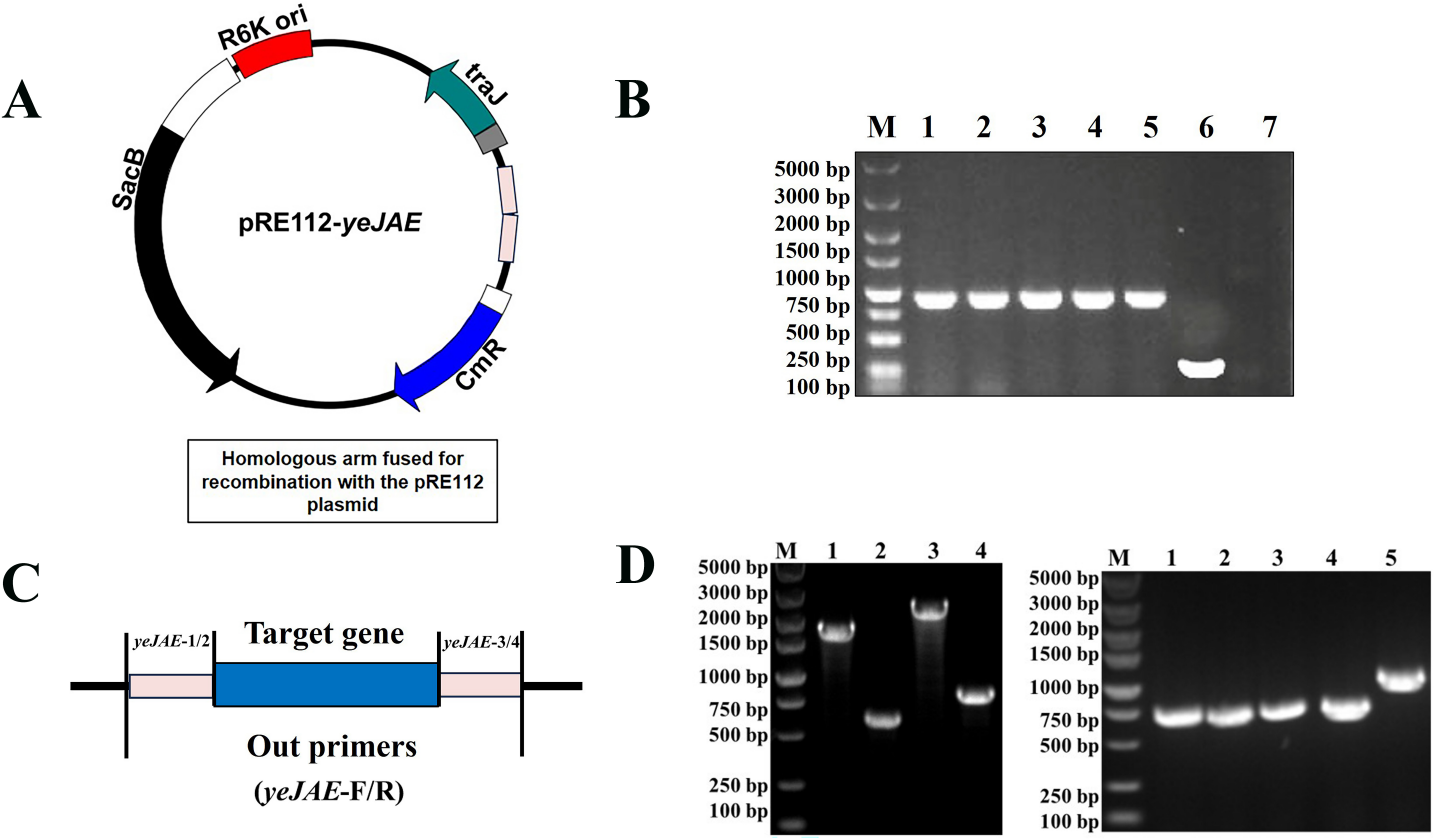
Construction and genetic verification of the RAST003 strain. **(A)** Schematic diagram of the pRE112-derived suicide plasmid constructed for deletion of the *yejAE* operon. The plasmid contains upstream and downstream homologous regions flanking the *yejAE* locus for allelic exchange. **(B)** PCR verification of the *yejAE* suicide plasmid. M: DL5000 DNA marker; Lane 1–5, PCR results for the pRE112- *yejAE* suicide plasmid. **(C)** Schematic representation of the locations of primers used for identification of gene deletions in the RAST003 strain. External primers were designed outside the homologous recombination regions to distinguish wild-type and deletion alleles. **(D)** PCR verification of gene deletions in the RAST003 strain. (a) PCR identification of the *manA* and *yejAE* deletions. M: DL5000 DNA marker; Lane 1–2: PCR results confirming successful deletion of the *manA* gene in the mutant strains; Lane 3–4: PCR results confirming successful deletion of the *yejAE* gene in the mutant strains (b) PCR identification of the *steD* deletion. M: DL5000 DNA marker; Lane 1–4: PCR results confirming successful deletion of the *steD* gene in the mutant strains; Lane 5: ATCC14028 wild-type strain used as a control.

To evaluate whether deletion of the *yejAE* operon, which has been implicated in modulating host immune responses, could further enhance CD8^+^ T cell activation in the attenuated *S*. Typhimurium vaccine RAST002, suicide plasmids targeting *yejAE* were constructed and a deletion mutant was generated using a SacB-based allelic exchange strategy (Figures 1A,B and Supplementary Figure 1). The deletion was verified by colony PCR with external primers flanking the homologous arms (Figures 1C,D and Supplementary Figures 2, 3), yielding a ∼2,381 bp fragment in the parental strain and a ∼790 bp fragment in the mutant, consistent with fusion of the upstream and downstream regions, and was further confirmed by Sanger sequencing. The resulting strain, designated RAST003, carries deletions of *manA*, *steD*, and *yejAE*. Safety was assessed following previously reported procedures by orally administering mice with bacterial suspensions up to 10^+^ CFU, and no deaths or adverse effects were observed, confirming the high attenuation of the strain (data not shown) (Zhou et al., 2025b).

### 3.2 Oral vaccination with RAST003 elicits strong antigen specific IgG and IgA antibody responses

**FIGURE 2:**
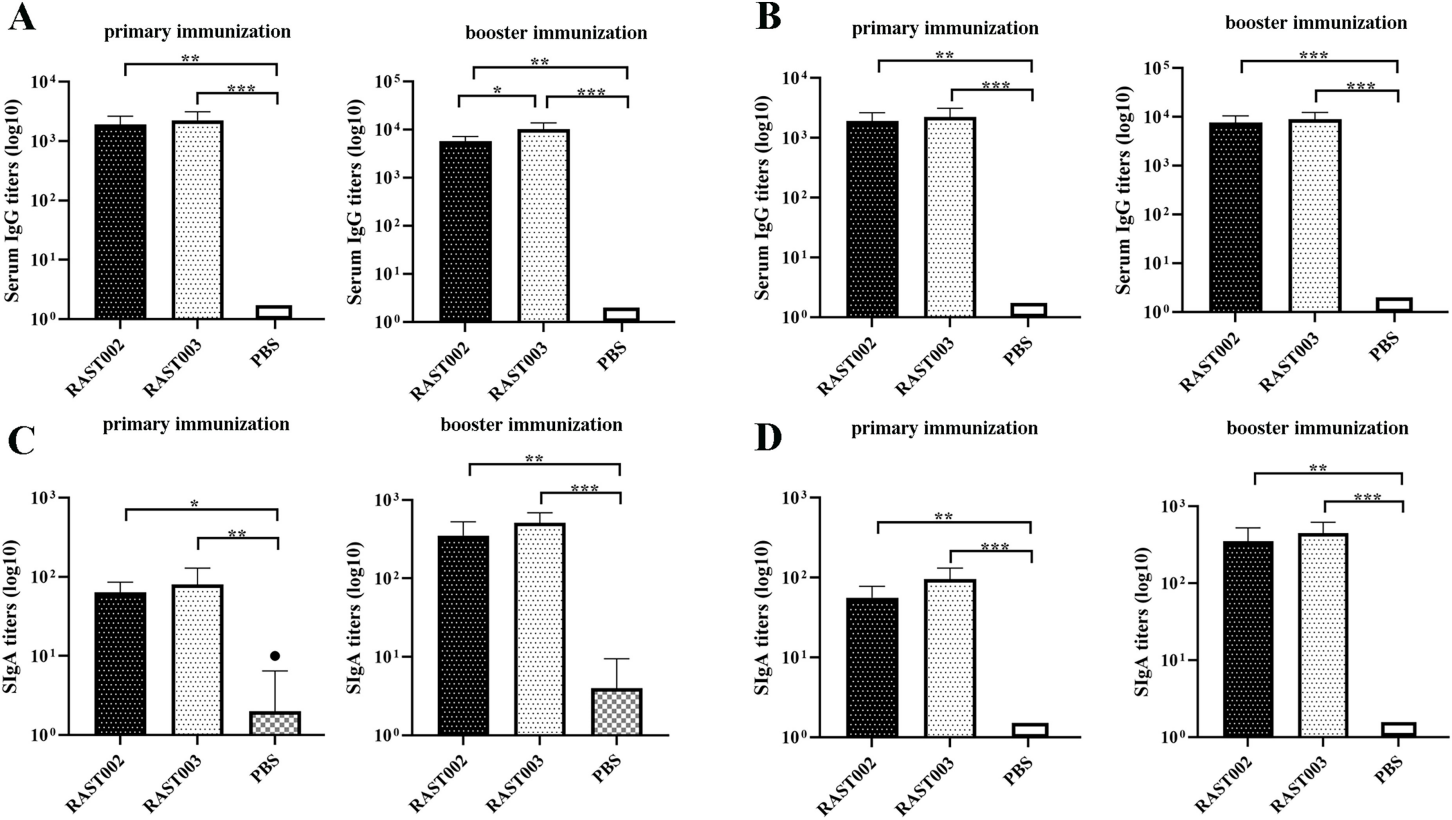
Serum IgG and mucosal IgA responses induced by RAST002 and RAST003. Serum and vaginal lavage samples were collected 7 days after the primary and booster immunizations. Antigen-specific antibody responses were measured by indirect ELISA using plates coated with outer membrane proteins from *S*. Typhimurium ATCC14028 (ST-OMPs) or *S*. Enteritidis (SE-OMPs). Serum IgG responses against ST-OMPs **(A)** and SE-OMPs **(B)** were determined to evaluate systemic immunity. Vaginal lavage IgA responses against ST-OMPs **(C)** and SE-OMPs **(D)** were measured to assess mucosal immunity. Data are presented as means ± SD (*n* = 5) from mice immunized with RAST002, RAST003, or PBS. Statistical significance is indicated as *p < 0.05, **p < 0.01, and ***p < 0.001 compared with PBS controls.

Specific systemic and mucosal antibody responses elicited by oral immunization were quantified by ELISA using serum and vaginal lavage samples collected at 7 days after the primary immunization and 7 days after the booster immunization. To assess cross-reactive antibody responses, plates were coated with either *S*. Typhimurium outer membrane proteins (ST-OMPs) or *S*. Enteritidis outer membrane proteins (SE-OMPs). As shown in Figures 2A,B, mice immunized with either RAST002 or RAST003 developed significantly elevated serum IgG responses compared with the PBS control group at all evaluated time points, regardless of the coating antigen. Notably, at 7 days after the booster immunization, mice vaccinated with the *yejAE* deficient strain RAST003 exhibited significantly higher IgG titers than both the PBS and RAST002 groups when ST-OMPs were used as the coating antigen (Figure 2A), indicating that deletion of *yejAE* further enhanced the recall humoral response against *S*. Typhimurium associated antigens. In contrast, under SE-OMPs coating conditions and at earlier sampling time points, serum IgG levels induced by RAST003 were comparable to those induced by RAST002, although both remained substantially higher than the PBS control (Figure 2B).

Mucosal immune responses were evaluated by measuring antigen specific sIgA in vaginal lavage samples. Consistent with the induction of systemic immunity, both RAST002 and RAST003 significantly increased sIgA levels relative to PBS controls at all-time points tested, irrespective of whether ST-OMPs or SE-OMPs were used for coating (Figures 2C,D). However, no statistically significant differences in sIgA titers were detected between the RAST002 and RAST003 groups under any experimental condition.

### 3.3 RAST003 Induces enhanced antigen-specific splenocyte proliferation

**FIGURE 3:**
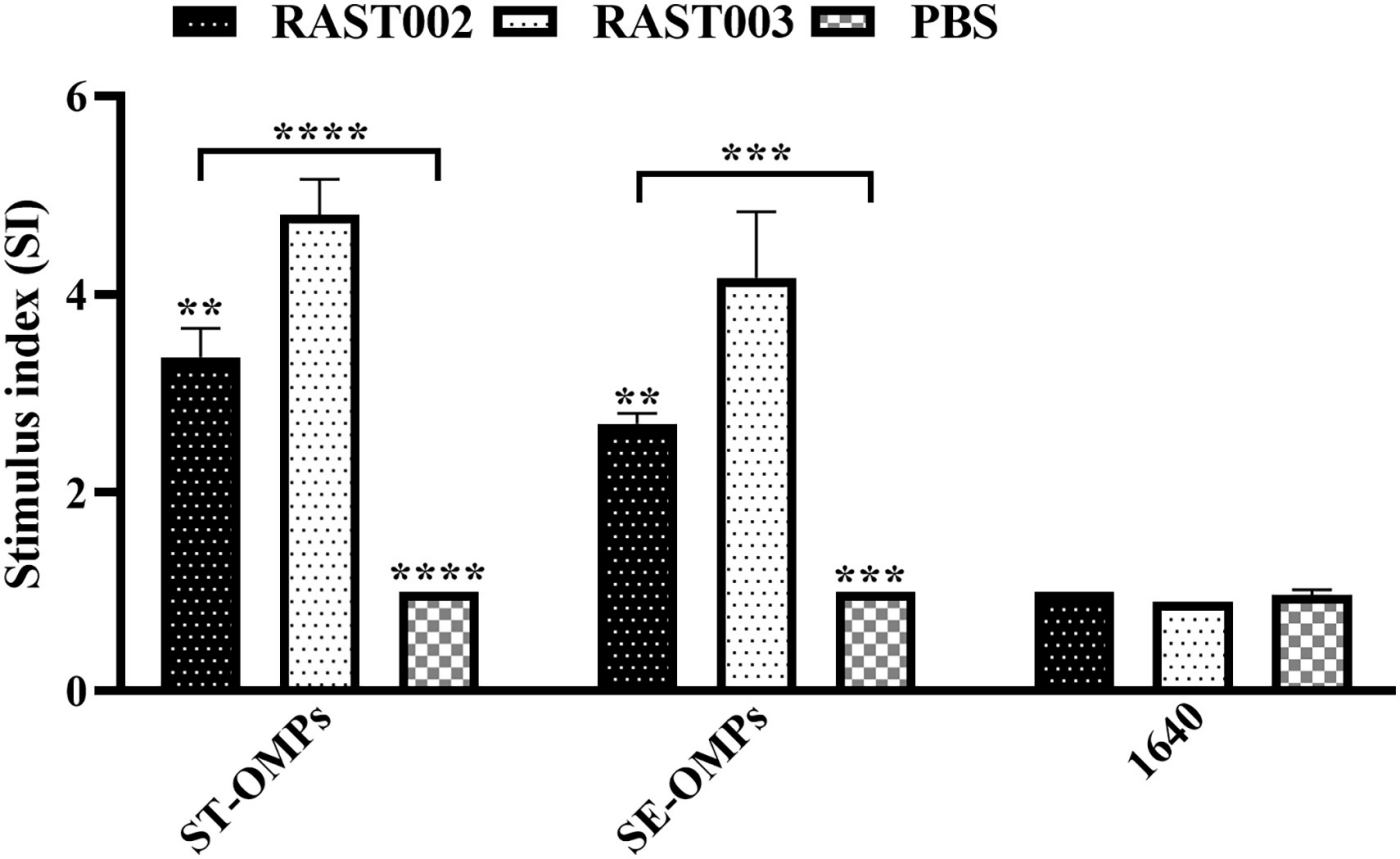
Antigen-specific splenocyte proliferation following vaccination. Splenocytes were collected 1 week after booster immunization from mice immunized with RAST002, RAST003, or PBS. Cells were stimulated *in vitro* with ST-OMPs or SE-OMPs, with RPMI 1640 medium serving as the negative control. Proliferative responses were measured using a CCK-8 assay, and stimulation indices were calculated. Data are presented as means ± SD (*n* = 3). Statistical significance is indicated as **p < 0.01, ***p < 0.001, and ****p < 0.0001 compared with PBS controls.

To evaluate antigen-specific cellular immune responses induced by oral immunization, splenocyte proliferation assays were performed 7 days after the booster immunization. Splenocytes isolated from immunized and control mice were stimulated *in vitro* with ST-OMPs or SE-OMPs, and cell proliferation was assessed using the CCK-8 assay. As shown in Figure 3, mice immunized with either RAST002 or RAST003 exhibited significantly enhanced proliferative responses compared with the PBS control group following stimulation with both antigens, indicating effective induction of antigen-specific cellular immunity. Importantly, splenocytes from mice immunized with RAST003, in which *yejAE* was additionally deleted in the RAST002 background, exhibited significantly higher stimulation indices than those from the RAST002 group under both ST-OMPs and SE-OMPs stimulation conditions, indicating that the additional deletion of *yejAE* further enhanced antigen-specific T cell responsiveness.

### 3.4 RAST003 enhances antigen-specific th1-biased cytokine responses

**FIGURE 4:**
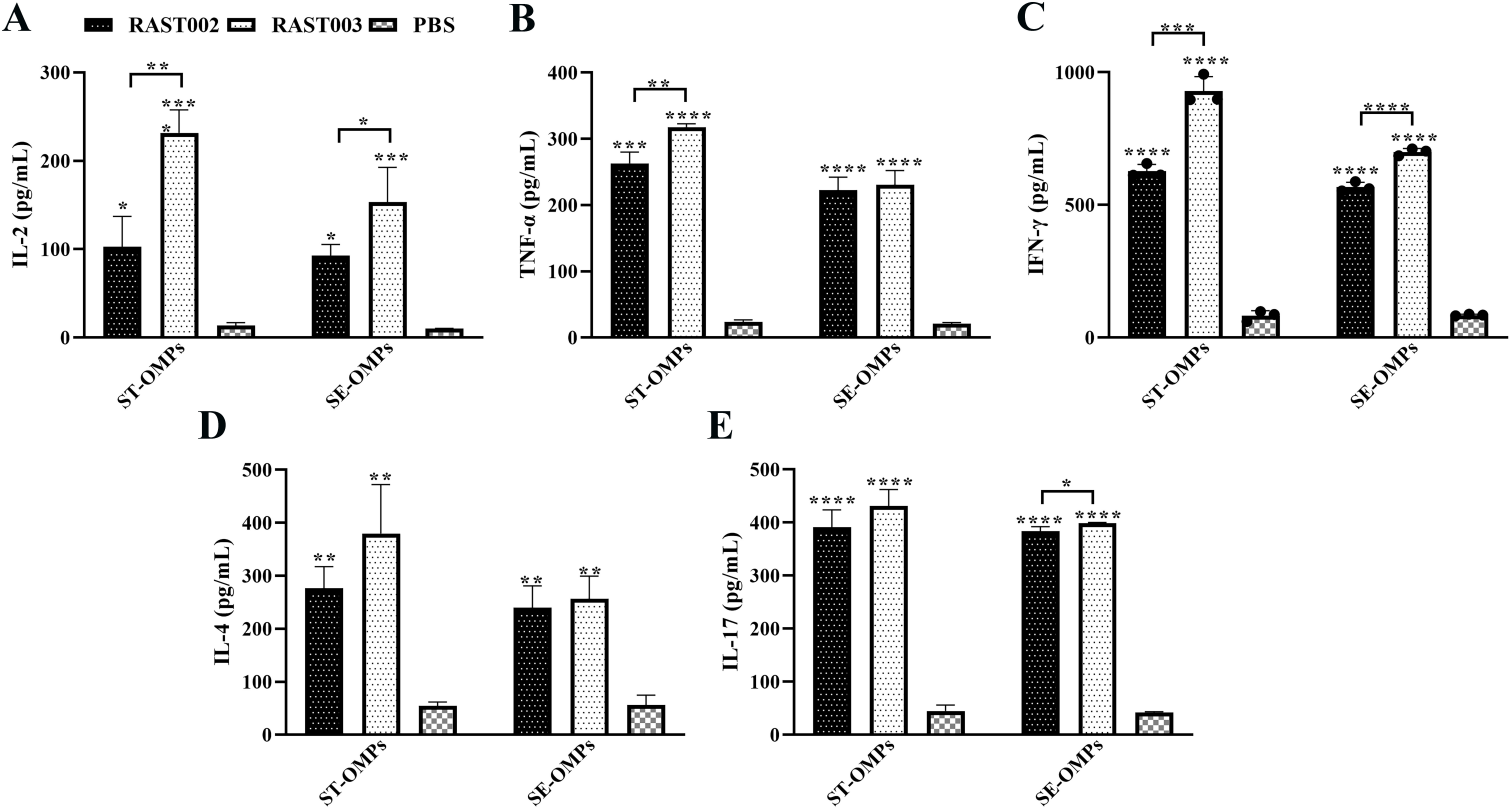
Antigen-specific cytokine responses induced by RAST002 and RAST003. Splenocytes were harvested 1 week after booster immunization from mice immunized with RAST002, RAST003, or PBS. Cells were stimulated *in vitro* with ST-OMPs or SE-OMPs, and culture supernatants were collected for cytokine determination. The concentrations of IL-2 **(A)**, TNF-α **(B)**, IFN-γ **(C)**, IL-4 **(D)**, and IL-17A **(E)** were measured by ELISA to evaluate vaccine-induced cellular immune responses. Data are presented as means ± SD (*n* = 5). Statistical significance is indicated as *p < 0.05, **p < 0.01, ***p < 0.001, and ****p < 0.0001 compared with PBS controls.

Cytokine secretion by splenocytes was quantified following *in vitro* antigen restimulation to evaluate vaccine-induced cellular immune responses. At 24 h, IL-2 and TNF-α secretion was significantly elevated in splenocytes from mice immunized with RAST002 or RAST003 compared with PBS controls, suggesting enhanced antigen-driven T cell activation and expansion (Figures 4A,B). Notably, the levels of IL-2 and TNF-α were significantly higher in the RAST003 group than in the RAST002 group, suggesting that the additional deletion of *yejAE* in the RAST002 background further enhanced T cell activation. At 72 h, IFN-γ production was significantly elevated in both vaccinated groups compared with the PBS controls, with RAST003 inducing significantly higher levels than RAST002 (Figure 4C). The increased IFN-γ production is indicative of a strong Th1-type cellular immune response. In addition, IL-4 levels were significantly increased in mice immunized with RAST002 or RAST003 compared with the PBS group (Figure 4D). However, no significant difference was observed between the two vaccinated groups, suggesting moderate induction of Th2-associated responses. Similarly, IL-17A levels were elevated in both vaccinated groups relative to PBS controls (Figure 4E). Moreover, SE-OMPs stimulation induced significantly higher IL-17A production in the RAST003 group than in the RAST002 group.

### 3.5 Enhanced CD8^+^ t cell responses induced by RAST003

**FIGURE 5:**
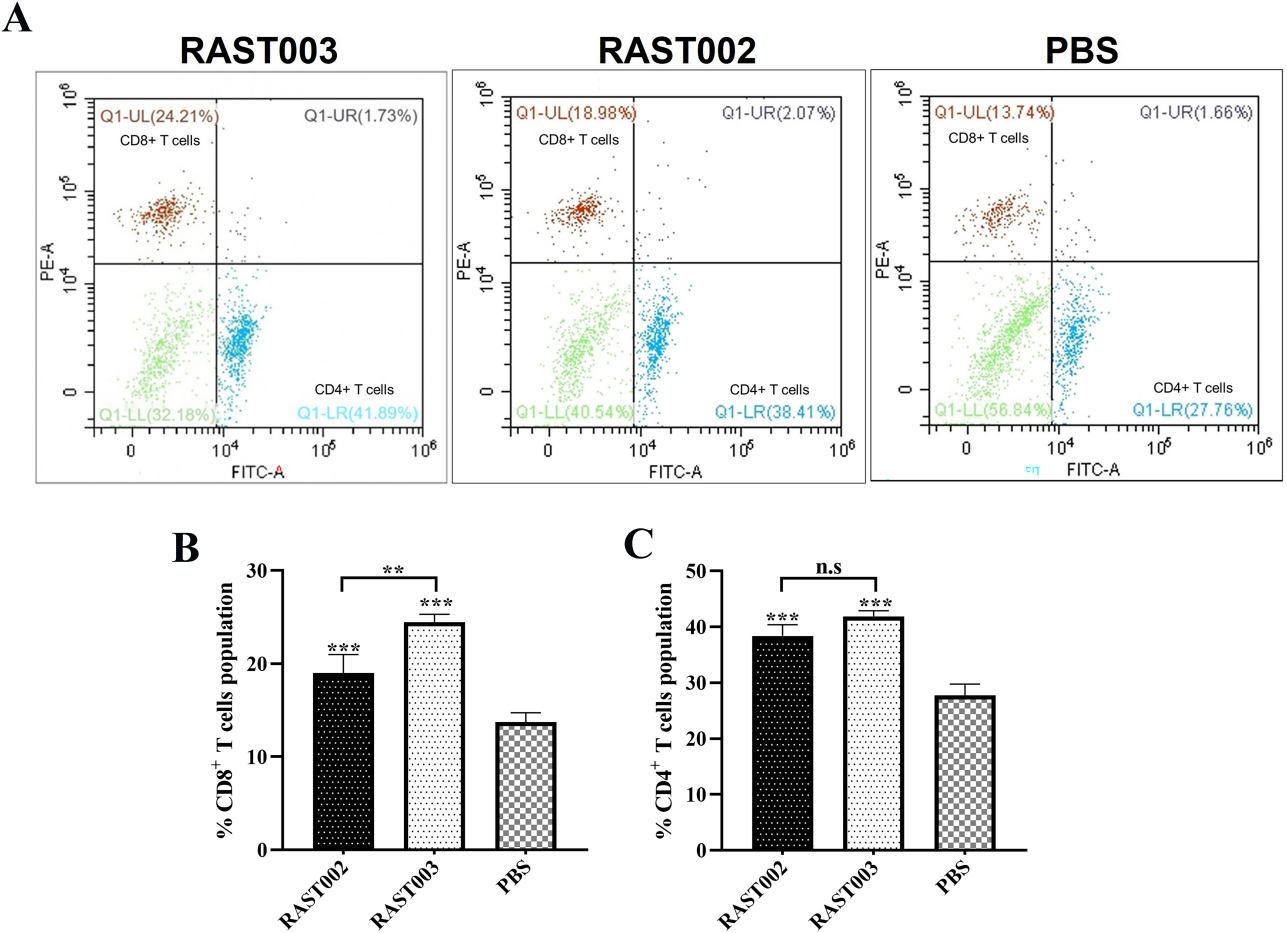
Frequencies of CD4^+^ and CD8^+^ T cell subsets following immunization with RAST002 and RAST003. **(A)** Representative flow cytometry plots showing the proportions of CD3^+^CD4^+^ and CD3^+^CD8^+^ T cells in splenocytes isolated from mice 1 week after booster immunization with RAST002, RAST003, or PBS. **(B)** Statistical analysis of CD3^+^CD8^+^ T cell frequencies across immunization groups. **(C)** Statistical analysis of CD3^+^CD4^+^ T cell frequencies across immunization groups. Data are presented as means ± SD (*n* = 3). Statistical comparisons were performed between groups. Significance is indicated by *p* < 0.01 (**) and *p* < 0.001 (***); n.s. indicates no significant difference.

The frequencies of CD4^+^ and CD8^+^ T cells were further analyzed by flow cytometry to directly assess T cell responses induced by vaccination (Figure 5A). Both RAST002 and RAST003 immunization significantly increased the proportion of splenic CD4^+^ and CD8^+^ T cells compared with the PBS control group, indicating effective induction of cellular immunity (Figures 5B,C). Notably, mice immunized with RAST003 displayed significantly higher frequencies of CD8^+^ T cells compared with those immunized with RAST002 (Figure 5B), whereas CD4^+^ T cell frequencies remained comparable between the two groups (Figure 5C). These findings suggest that the additional deletion of *yejAE* in the RAST002 background preferentially promotes CD8^+^ T cell expansion, consistent with the enhanced Th1-associated cytokine responses observed in RAST003-immunized mice.

### 3.6 RAST003 confers complete homologous protection and enhanced cross-serotype survival

**FIGURE 6:**
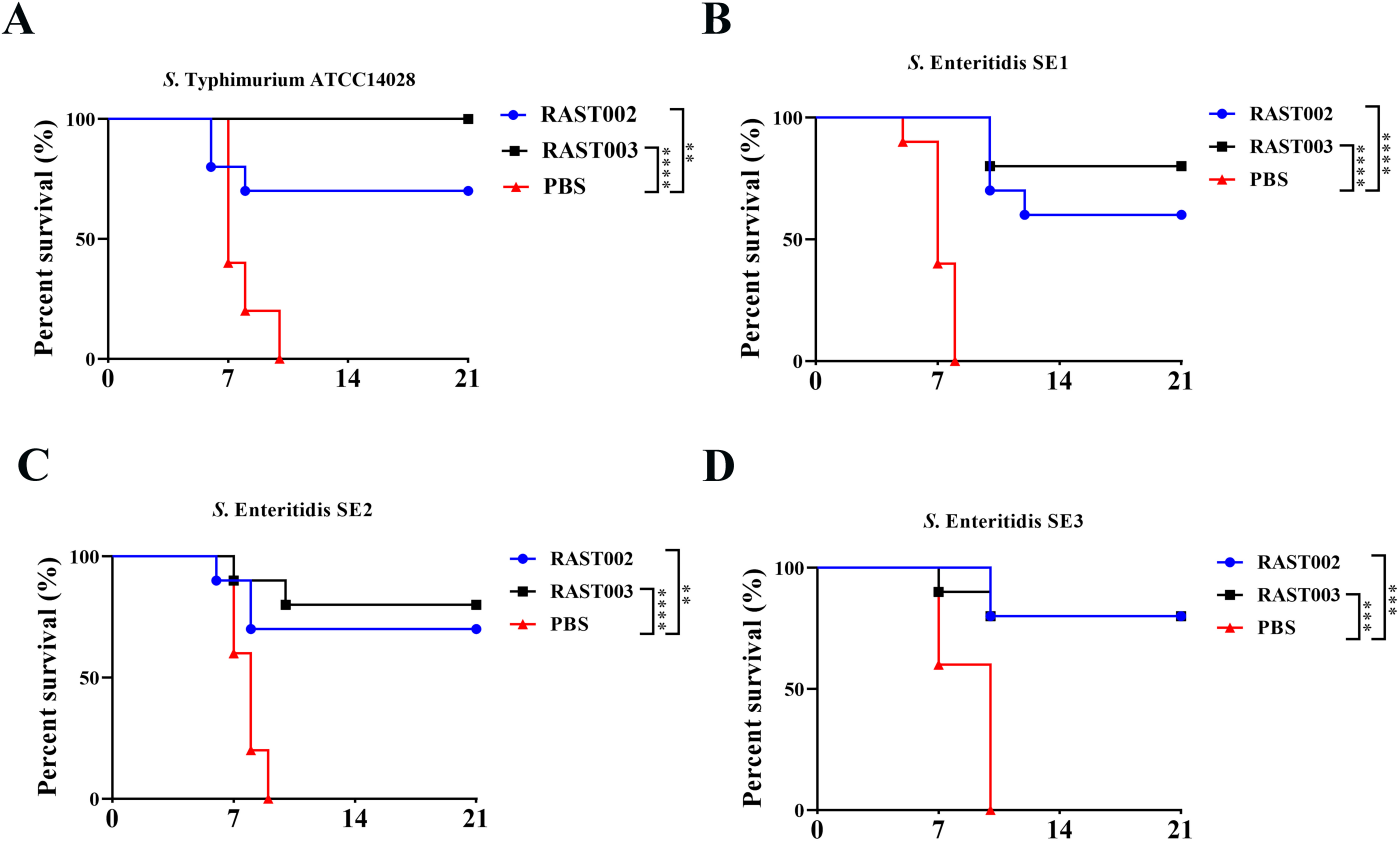
Protective efficacy of RAST002 and RAST003 against homologous and heterologous *Salmonella* infection. Three weeks after booster immunization, mice vaccinated with RAST002, RAST003, or PBS were orally challenged with lethal doses of *S*. Typhimurium ATCC14028 **(A)** or field isolates of *S*. Enteritidis SE1 **(B)**, SE2 **(C)**, and SE3 **(D)**. Survival was monitored daily for 21 days post challenge. Kaplan–Meier survival curves are shown for each group. Statistical significance is indicated as *p* < 0.01 (**), *p* < 0.001 (***), and *p* < 0.0001 (****).

Protective efficacy was evaluated by monitoring survival following oral challenge with ∼100 × LD_50_ of wild-type *S*. Typhimurium ATCC14028 and field-isolated *S*. Enteritidis strains (SE1, SE2, and SE3). Consistent with our previous findings, immunization with RAST002 conferred substantial protection, with survival rates comparable to those observed in earlier experiments. In contrast, RAST003 immunization resulted in complete protection against homologous *S*. Typhimurium ATCC14028 challenge, with 100% of mice surviving throughout the 21-day observation period (Figure 6A). Following challenge with the heterologous *S*. Enteritidis strains, RAST003 provided 80% protection across all three isolates (Figures 6B–D). The current results demonstrate superior performance against both homologous and heterologous serotypes. In all cases, mice in the PBS control group succumbed to infection within 14 days.

## 4 Discussion

Live attenuated vaccines against intracellular bacteria must strike a delicate balance between safety and immunogenicity (Rasko and Sperandio, 2010; Riquelme et al., 2011). While attenuation reduces virulence, excessive reduction of bacterial fitness or immune activation can compromise protective efficacy (Rasko and Sperandio, 2010; Zhou et al., 2023). In this study, we sought not merely to attenuate *Salmonella*, but to rationally reconfigure host-pathogen interactions by systematically dismantling bacterial immune evasion mechanisms. Our findings demonstrate that concurrent deletion of *steD* and *yejAE* in an LPS-deficient background reshapes antigen presentation dynamics and substantially enhances protective immunity.

Effective immunity against *Salmonella* relies predominantly on Th1-skewed cellular responses, particularly IFN-γ dependent macrophage activation and cytotoxic CD8^+^ T cell activity (Zhou et al., 2023). Previous studies established that the *yej* operon interferes with MHC class I antigen presentation, thereby limiting CD8^+^ T cell priming (Qimron et al., 2004). By eliminating *yejAE*, we observed a marked amplification of antigen-specific CD8^+^ T cell frequencies and increased production of IL-2, TNF-α, and IFN-γ. Importantly, this enhancement was selective rather than indiscriminate, as the cytokine profile remained Th1-dominant without evidence of excessive inflammatory escalation. These findings suggest that removal of *yejAE* restores productive MHC-I dependent antigen presentation, thereby reinforcing cytotoxic T cell mediated control of intracellular infection.

The immunological gains observed with RAST003 were not restricted to cellular responses. LPS deficiency likely increases exposure of conserved outer membrane antigens, facilitating cross-reactive antibody responses, while deletion of *steD* alleviates MHC II-associated suppression and supports effective CD4^+^ T cell help (Bayer-Santos et al., 2016; Domínguez-Medina et al., 2020; Cerny et al., 2021; Godlee et al., 2022). The integration of these modifications resulted in coordinated activation of both humoral and cellular arms of adaptive immunity. Such balanced immune induction appears critical, as RAST003 achieved complete protection against homologous challenge and sustained high-level protection against heterologous serovars.

These findings support a model wherein the coordinated effects of structural antigen unmasking and restored antigen presentation pathways operate synergistically to enhance immunogenicity (Allen et al., 2024; Zhou et al., 2025b; Zhou et al., 2025a). Rather than relying solely on attenuation, this approach involves the targeted removal of specific immune interference mechanisms encoded within the bacterial genome, thereby enabling the host immune system to generate a more robust and appropriately polarized response. The sequential deletion of genes responsible for LPS-mediated shielding, MHC II interference (*steD*), and MHC I modulation (*yejAE*) reflects a rational, systems-level strategy for vaccine design.

Together, these observations have broader implications for the design of live vaccines against intracellular pathogens. Many bacterial species deploy conserved mechanisms to dampen antigen presentation and T cell activation (Teymournejad and Montgomery, 2021; Cheng et al., 2024). Targeted disruption of such pathways, combined with controlled attenuation, may provide a generalizable framework for enhancing vaccine-induced cellular immunity while maintaining safety. Future studies are needed to define the durability of memory responses, assess translational performance in relevant livestock, and apply integrated multi-omics approaches to guide vaccine development (Tian et al., 2026; Zhou and Hu, 2026).

In conclusion, our study demonstrates that rational dismantling of immune evasion strategies can transform an attenuated strain into an immunologically optimized vaccine platform. This work advances a conceptual shift from empirical attenuation toward mechanism-guided vaccine engineering.

## Data Availability

The raw data supporting the conclusions of this article will be made available by the authors, without undue reservation.
