# Supplementary figures and images for: Deletion of steD gene and yejAE operons in LPS-deficient Salmonella elicits strong t cell immunity and complete protection against homologous serotypes
Source: Front Microbiol. 2026 Jun 17;17:1827967. doi: 10.3389/fmicb.2026.1827967 (PMC13319094; doi:10.3389/fmicb.2026.1827967)

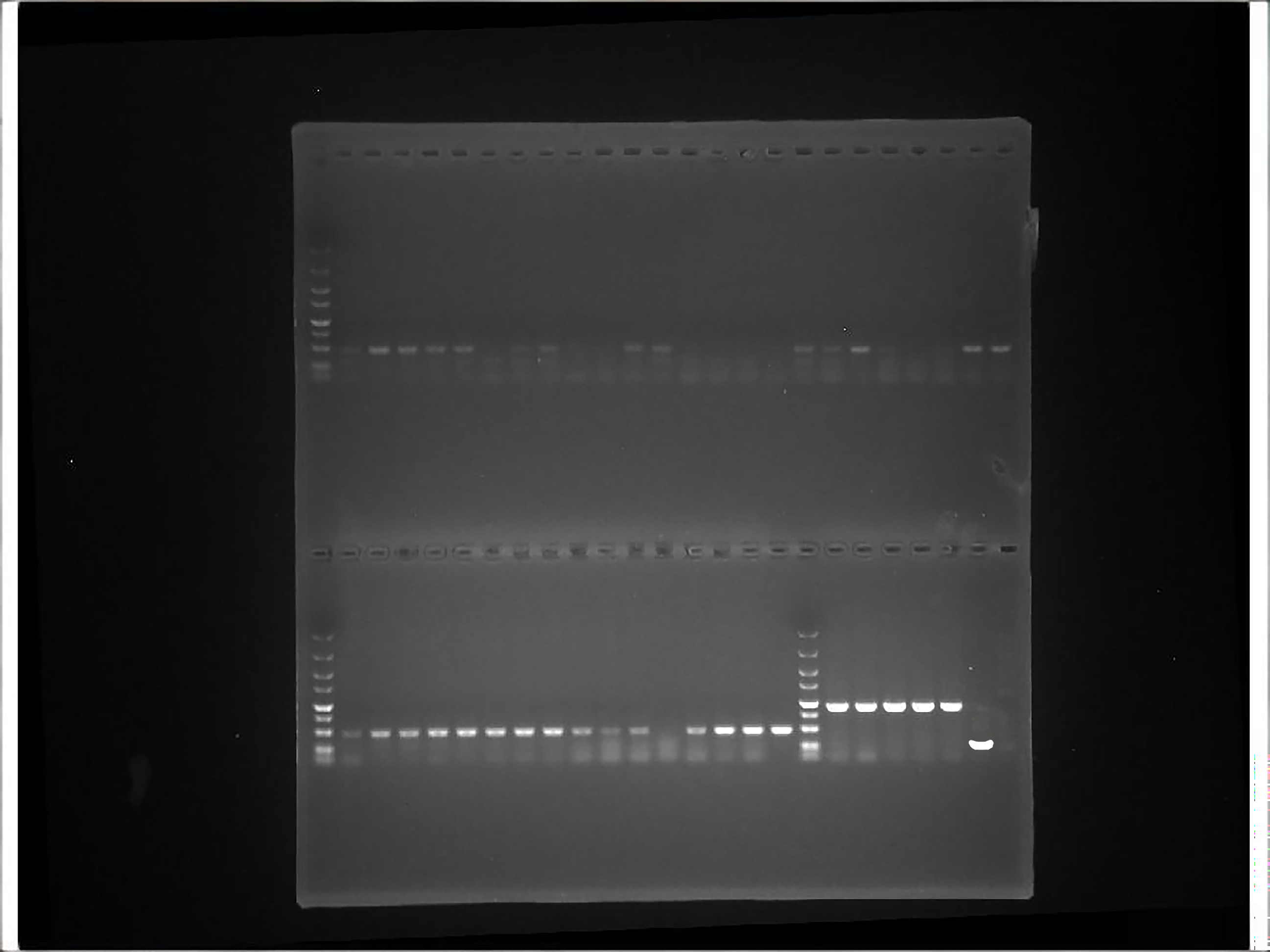

Supplement: Supplementary file 1 [file Data_Sheet_1.zip › original gel images/Fig.S1.tif]

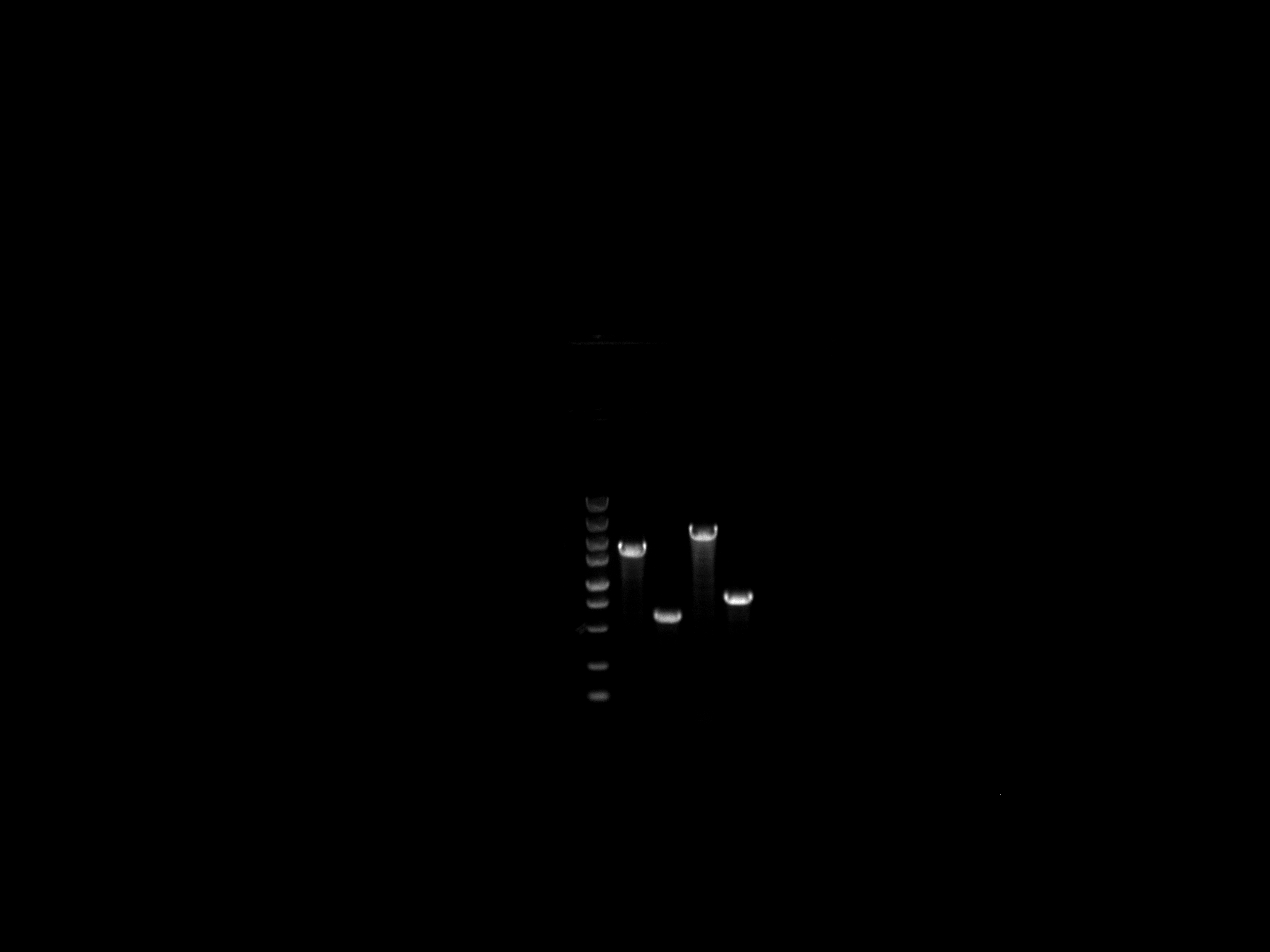

Supplement: Supplementary file 1 [file Data_Sheet_1.zip › original gel images/Fig.S2.tif]

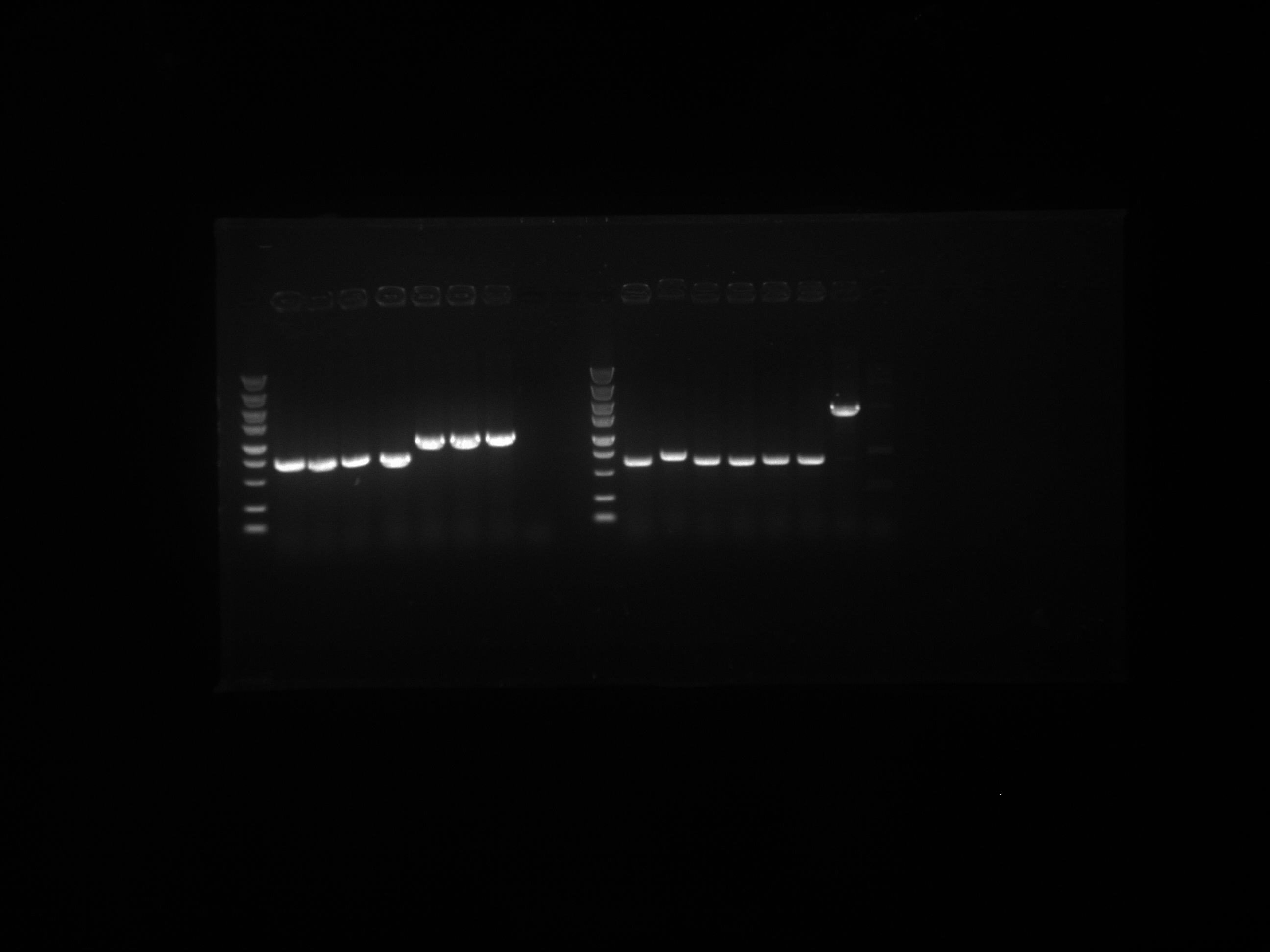

Supplement: Supplementary file 1 [file Data_Sheet_1.zip › original gel images/Fig.S3.tif]
